# Pneumomediastinum After Third Molar Surgery: A Case Report

**DOI:** 10.7759/cureus.112649

**Published:** 2026-07-14

**Authors:** Carlos Ulloa Luchsinger, Sebastián Tapia Corón, Hernán Ramírez Skinner

**Affiliations:** 1 Faculty of Dentistry, Pontificia Universidad Católica de Chile, Santiago, CHL

**Keywords:** air-driven handpiece, case report, cervicofacial emphysema, dental complication, oral surgery, pneumomediastinum, subcutaneous emphysema, third molar extraction

## Abstract

Cervicofacial emphysema is defined as the pathological presence of air within the fascial spaces of the neck and face. Dental procedures are a recognized but uncommon cause, and only rarely do they involve the mediastinum; such cases are typically related to the use of high-speed air-driven handpieces or air/water (triple) syringes, producing pneumomediastinum. We report a 22-year-old woman who presented to the emergency department with sudden-onset dyspnea immediately after the extraction of two right-sided third molars performed with high-speed air-driven rotary instruments. Contrast-enhanced computed tomography confirmed cervical subcutaneous emphysema and pneumomediastinum. She was admitted for airway and hemodynamic monitoring; an esophagogram excluded esophageal injury, prophylactic antibiotics were administered, and she recovered uneventfully, being discharged on the third day. At one-week follow-up, the patient reported complete resolution of dyspnea and chest pain, with no residual cervical swelling or crepitus. Although air-driven handpieces and air syringes are safe and essential tools in dental practice, their use during invasive procedures and in anatomically high-risk areas warrants caution, as it may rarely result in this potentially life-threatening complication. Early recognition and a conservative, multidisciplinary approach are key to a favorable outcome.

## Introduction

Third molar extraction is one of the most frequently performed oral surgical procedures, with reported complication rates ranging from 3.5% to 14%, most of which are minor and self-limiting. Surgical site infection, pain, oroantral communication, hemorrhage, bone sequestra, alveolar osteitis, and hypoesthesia are among the most prevalent complications associated with this procedure [1].

A rare but potentially life-threatening complication is cervicofacial emphysema (CFE): the pathological presence of air within the soft tissues of the neck and face. While it may be a common sign in conditions such as facial fractures, wounds, infections, or following head and neck surgery, CFE after a dental procedure is unusual. It is associated mainly with the use of high-speed air-driven rotary instruments (turbines) and/or compressed-air syringes (triple syringes) during invasive procedures, in which the propelled air finds a path of least resistance, dissecting cervical spaces and, on rare occasions, compromising thoracic structures such as the mediastinum [2]. Notably, routine non-surgical dental procedures rarely cause CFE because epithelial integrity remains intact; in contrast, oral surgical procedures create intentional epithelial discontinuity through flaps and incisions, providing a direct channel for pressurized air to enter the deeper cervical fascial spaces. The objective of this report is to describe a rarely encountered case of pneumomediastinum following third molar extraction, to raise awareness of this uncommon but potentially life-threatening complication and its management; the case is reported in line with the CARE guidelines.

## Case presentation

A 22-year-old woman with no relevant medical history and no known allergies developed sudden-onset dyspnea and retrosternal pain radiating to the head and neck during the extraction of two right-sided third molars (one maxillary and one mandibular), performed at a facility outside our healthcare network. She presented to our emergency department because of dyspnea that had begun during the procedure and worsened over the following four hours.

On physical examination, she was normotensive (133/74 mmHg), with a normal heart rate (68 bpm), no oxygen requirement (oxygen saturation 98%), and afebrile (axillary temperature 36.3°C). Cervical examination revealed diffuse swelling with palpable crepitus extending from the left temporal region, across the bilateral submandibular areas, and down the ventral neck to the sternal region. The right maxillary and mandibular third molars were absent, with alveolar sockets covered by a stable clot.

**Figure 1:**
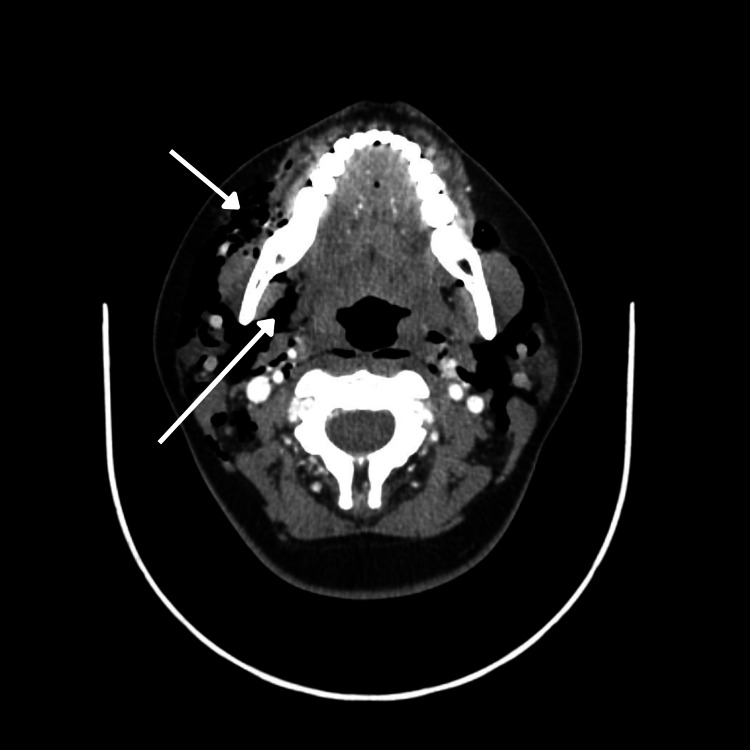
Axial contrast-enhanced CT at the level of the mandible and oropharynx Gas (arrows) dissects the bilateral masticator, buccal, parapharyngeal, and submandibular spaces. CT: computed tomography

**Figure 2:**
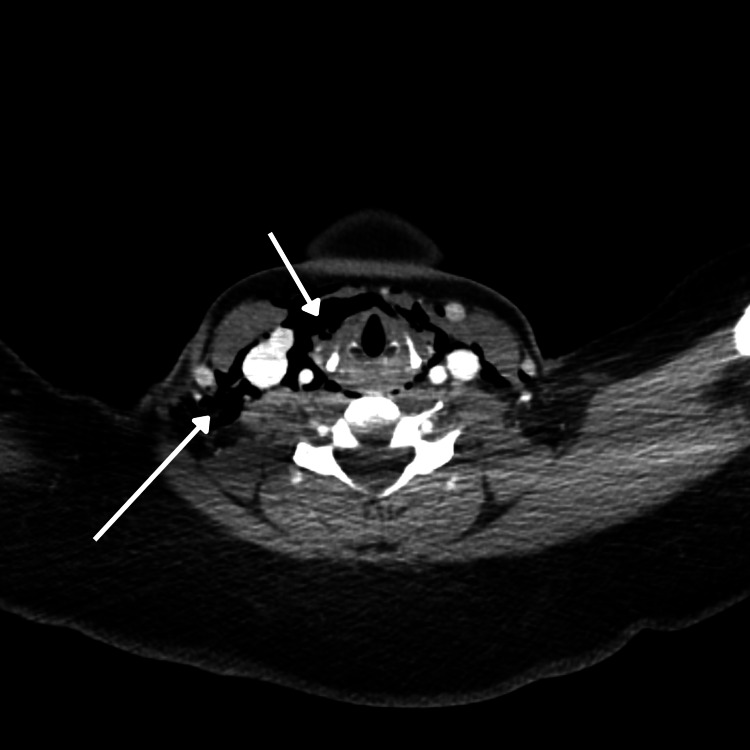
Axial contrast-enhanced CT of the thorax Pneumomediastinum, with gas (arrow) surrounding the trachea and the mediastinal great vessels, alongside anterior chest-wall subcutaneous emphysema. CT: computed tomography

**Figure 3:**
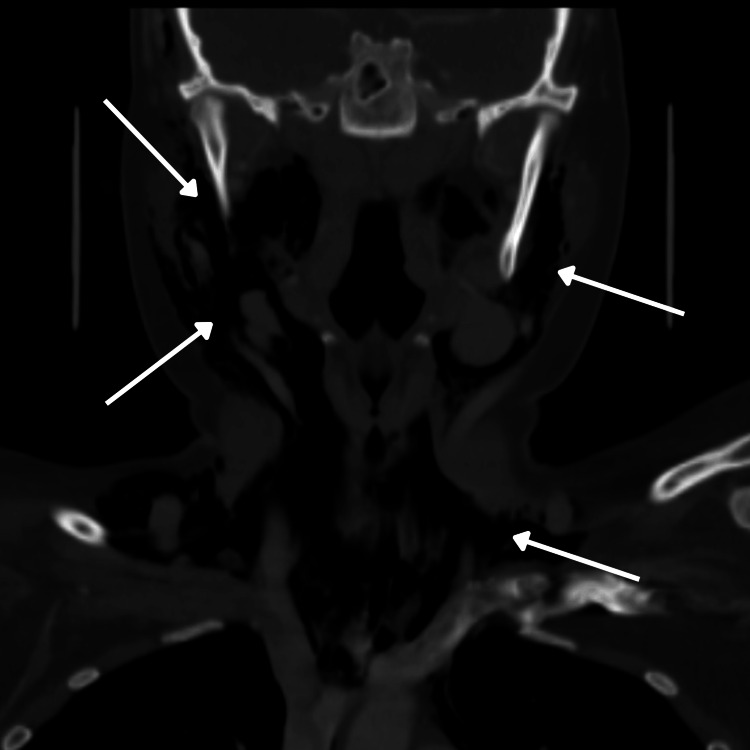
Coronal contrast-enhanced CT of the neck and thorax Gas (arrows) extends in continuity from the cervicofacial soft tissues through the thoracic inlet into the mediastinum. CT: computed tomography

**Figure 4:**
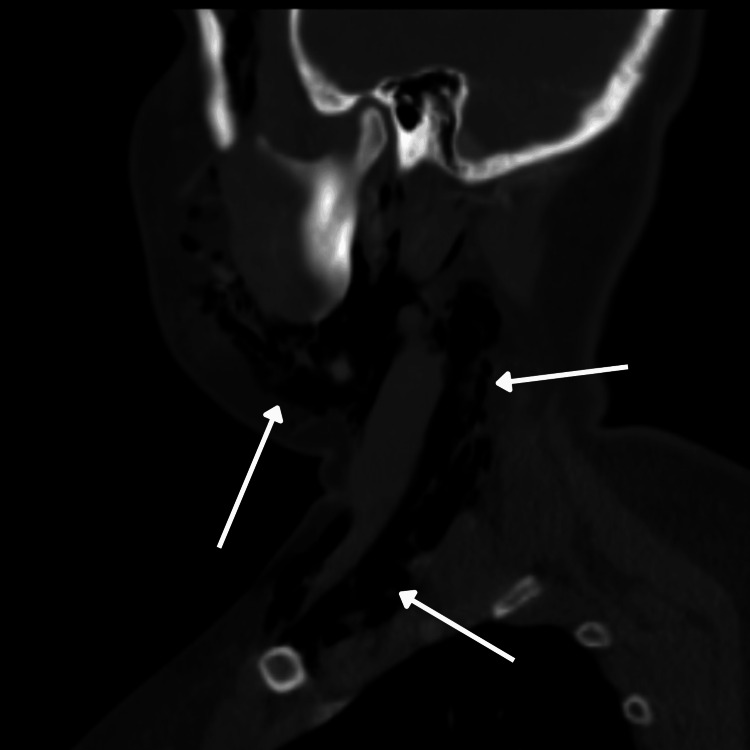
Sagittal contrast-enhanced CT of the neck and thorax A column of gas (arrows) tracks from the submandibular region along the anterior neck into the anterior and superior mediastinum. CT: computed tomography

Contrast-enhanced computed tomography (CT) of the neck and chest demonstrated gas involving the bilateral masticator spaces and extending to the temporal spaces bilaterally at its superior margin (Figure 1). From both masticator spaces, the air dissected from superficial to deep planes (through the pterygomandibular, parotid, and parapharyngeal spaces to the retropharyngeal space), which, in a cephalocaudal direction, involved the mediastinum, surrounding the trachea, heart, and supra-aortic vessels, reaching the diaphragm at its inferior limit (Figures 2-4). Extensive subcutaneous emphysema was also noted, extending from the submandibular region to the bilateral supraclavicular region.

A diagnosis of cervical subcutaneous emphysema and pneumomediastinum was confirmed, and the patient was admitted for airway and hemodynamic monitoring. The case was managed jointly with the thoracic surgery team, and prophylactic antibiotic therapy with ampicillin/sulbactam was started. During admission, a contrast esophagogram was performed to rule out esophageal injury; no abnormalities were found. Chest radiographs at 24 and 48 hours confirmed the stability of the pneumomediastinum. Once other probable causes had been excluded and based on a directed history, the principal suspected etiology was the use of compressed-air-driven rotary instruments during the third molar extraction.

The patient progressed favorably (hemodynamically stable, normotensive, with a normal heart rate, and without respiratory or infectious compromise) and was discharged on the third day with early outpatient follow-up. Oral antibiotic therapy with amoxicillin/clavulanic acid 875/125 mg every 12 hours and analgesics were maintained for one week. At the seven-day follow-up, the patient reported complete resolution of dyspnea and chest pain, with no residual cervical swelling or crepitus, and was discharged from care. The clinical course is summarized in Table 1.

**Table 1:** Clinical timeline (referenced to the dental procedure, Day 0). CT: computed tomography

Time point	Event
Day 0 (procedure)	Extraction of the right maxillary and mandibular third molars with a high-speed air-driven handpiece; sudden-onset dyspnea and retrosternal pain began during the procedure.
Day 0, +4 hours	Progressive dyspnea; presentation to the emergency department. Normotensive, afebrile, oxygen saturation 98%, diffuse cervicofacial swelling with crepitus.
Day 0	Contrast-enhanced CT: bilateral cervicofacial subcutaneous emphysema and pneumomediastinum. Admission; joint management with thoracic surgery; intravenous ampicillin/sulbactam.
Day 1	Contrast esophagogram: no esophageal injury. Chest radiograph at 24 h: stable.
Day 2	Chest radiograph at 48 h: stable; clinically improving.
Day 3	Discharged on oral amoxicillin/clavulanic acid 875/125 mg every 12 h and analgesia for one week.
Day 7	Outpatient follow-up: asymptomatic; discharged from care.

## Discussion

Air within the soft tissues of the head and neck can arise through several mechanisms [2]. In the dental setting, the cause is usually iatrogenic, related to compressed-air instruments; however, air emphysema of the oral and perioral regions has also been described after non-iatrogenic increases in intraoral pressure (such as playing wind instruments, forceful Valsalva maneuvers, or coughing), after facial trauma, and in the context of infection by gas-forming organisms. Indeed, the first reported case of this entity, more than a century ago, occurred in a patient who developed subcutaneous emphysema after playing a wind instrument following a dental extraction. In the iatrogenic form, pressurized air introduced at the operative site follows the path of least resistance along contiguous fascial planes, producing cervicofacial subcutaneous emphysema and, when the air dissects caudally, pneumomediastinum [2]. What makes the present case unusual is not the mechanism itself but its extent: air originating from a third molar extraction dissected from the masticator spaces to the mediastinum, an uncommon and potentially life-threatening progression.

Pneumomediastinum is defined as the presence of air within the mediastinal interstitium, which may, on rare occasions, originate during or after a dental procedure. The clinical picture is characterized by swelling, a sensation of pressure in the facial/cervical region, crepitus, erythema, dysphagia, dysphonia, dyspnea, and pain, including retrosternal pain [2,3]. Physical examination complemented by CT confirms the diagnosis, and imaging is essential. The differential diagnosis includes hypersensitivity reaction, hematoma, cellulitis, angioedema, and acute coronary syndrome [2].

Four basic routes have been described by which air from extramediastinal structures may enter the mediastinum: (I) through the fascial planes of the neck; (II) via tracheal, bronchial, or esophageal perforation; (III) from the retroperitoneal space through the diaphragmatic hiatus; and (IV) directly from the lungs [4].

Air-driven rotary instruments (turbines) play a fundamental role in dental practice and are used widely and very safely. They are powered by compressed air at 3.5-4.0 kgf/cm² and rotate at up to approximately 450,000 rpm; water sprays cool the heat generated by the rotary elements (burs) and wash away enamel and dentin debris [5].

Arai et al. [5] analyzed 47 cases of subcutaneous emphysema and pneumomediastinum due to a dental procedure reported between 1994 and 2008. In the 45 patients with available treatment details, 18 developed the complication after a dental extraction, 15 after a restoration, four after endodontic treatment, three after periodontal treatment, two after an apicoectomy, and three after other procedures. Among those undergoing extractions, as in our patient, most cases occurred after third molar extraction in which the tooth was sectioned using an air-driven turbine, allowing air to enter the parapharyngeal space through the mucosal incision. CFE has also been described after less invasive procedures without mucosal incisions, suggesting that air from turbines can find a path of least resistance, such as a periodontal pocket or the root canal system.

Jones et al. [6] published a systematic review of subcutaneous emphysema following dental procedures between 1993 and 2020, including 135 cases. A large proportion (54%) were associated with dental extractions, most often of mandibular molars; the majority were iatrogenic, with 51% attributed to an air-driven handpiece and 9% to air syringes. The mediastinum was the most frequently involved compartment beyond the cervicofacial soft tissues.

More recent reports confirm that this complication continues to occur and underscore its potential severity. Peters et al. [7] described a further case of pneumomediastinum after third molar extraction with an updated literature review, and Sepsick et al. [8] reported a severe case complicated by mediastinitis and deep neck and mediastinal abscesses after extraction with a high-speed air-driven handpiece, requiring surgical incision and drainage and video-assisted thoracoscopic surgery. These cases reinforce that, although most patients recover with conservative management, life-threatening progression remains possible.

Although pneumomediastinum usually resolves spontaneously within the first 10 days, potential complications include mediastinitis, cardiac tamponade, airway obstruction, simple or tension pneumothorax, pneumoperitoneum, infection (cervical necrotizing fasciitis with descending mediastinitis), and even death from sepsis or air embolism [9-11]. Early recognition is essential to prevent infectious complications arising from the spread of oral flora into deep cervical spaces, and antibiotic therapy covering oral flora is recommended [12]. In severe cases, surgical decompression may be required [2]. Most patients experience mild to moderate local complications; rarely, principally when mandibular molars are involved, upper-airway compromise may occur owing to the communication of these spaces with the sublingual, submandibular, parapharyngeal, and retropharyngeal spaces [11].

This report has limitations. As a single case, it cannot establish causation or incidence, and the etiology is presumptive, inferred from the immediate temporal association with the use of an air-driven handpiece, a directed history, and exclusion of alternative causes. As the index procedure was performed at an external facility, intraoperative technical details could not be independently verified.

## Conclusions

Compressed-air-powered dental instruments are widely used and generally safe; however, their use during invasive procedures may rarely result in cervicofacial emphysema, with extension of air into deep cervical fascial spaces and, in rare cases, the mediastinum. Conventional air-driven rotary handpieces, which exhaust compressed air into the operative field, should not be used for oral surgical procedures such as osteotomies or tooth sectioning; where mechanical cutting is required, surgical handpieces that vent air away from the field or electric micromotors are preferred. Particular caution should also be exercised in anatomically high-risk areas, such as the root canal system during endodontic access or near a periodontal pocket during finishing of a cervical restoration. Prompt recognition, appropriate imaging, close clinical monitoring, and multidisciplinary management were associated with a favorable outcome in this patient and are supported by the available literature. Most reported cases resolve successfully with conservative multidisciplinary management when life-threatening causes have been excluded and close monitoring is provided.
